# Sudden vision loss and neurological deficits after facial hyaluronic acid filler injection

**DOI:** 10.1186/s42466-022-00203-x

**Published:** 2022-07-18

**Authors:** Alexandra Lucaciu, Patrick Felix Samp, Elke Hattingen, Roxane-Isabelle Kestner, Petra Davidova, Thomas Kohnen, Jasmin Rudolph, Andreas Dietz, Helmuth Steinmetz, Adam Strzelczyk

**Affiliations:** 1 Department of Neurology, Center of Neurology and Neurosurgery, University Hospital and Goethe-University Frankfurt, Schleusenweg 2-16, 60528 Frankfurt am Main, Germany; 2 Department of Neuroradiology, University Hospital and Goethe-University Frankfurt, Frankfurt am Main, Germany; 3 Department of Ophthalmology, University Hospital and Goethe-University Frankfurt, Frankfurt am Main, Germany; 4 Department of Ear, Nose and Throat Surgery, University of Leipzig, Leipzig, Germany

**Keywords:** Hyaluronic acid filler injection, Glabella, Eye, Ptosis, Blindness, Embolism, Vascular obstruction, Neurological deficit, Ischemic optic neuropathy, Necrosis

## Abstract

**Background:**

The ongoing expansion of the cosmetic armamentarium of facial rejuvenation fails to uncover the inherent risks of cosmetic interventions. Informed consent to all risks of cosmetic filler injections and potential sequelae, including ocular and neurological complications, should be carefully ensured. We present two cases of complications following facial hyaluronic acid filler injections.

**Case presentations:**

Case 1: A 43-year-old woman presented with monocular vision loss of the left eye, associated ptosis, ophthalmoplegia, periocular pain and nausea, cutaneous changes of the glabella region and forehead, and sensory impairment in the left maxillary branch dermatome (V2) after receiving a hyaluronic acid (HA) filler injection into the left glabellar area. On ophthalmological examination, an ophthalmic artery occlusion (OAO) was diagnosed upon identification of a “cherry-red spot”. Magnetic resonance imaging (MRI) revealed a left ischemic optic neuropathy. Supportive therapy and hyaluronidase injections were initiated. A follow-up MRI of the head performed two months after presentation corresponded to stable MRI findings. The patient had irreversible and complete vision loss of the left eye, however, the ptosis resolved.

Case 2: A 29-year-old woman was admitted to hospital a few hours after a rhinoplasty and cheek augmentation with hyaluronic acid, presenting with acute monocular vision loss in the right eye, retrobulbar pain, fatigue and vomiting. In addition, the patient presented a harbinger of impending skin necrosis and a complete oculomotor nerve palsy on the right side, choroidal ischemia and vision impairment. Supportive treatment and hyaluronidase injections into the ischemic tissue were initiated. A small scar at the tip of the nose, vision impairment and an irregular pupillary margin on the right side persisted at follow-up.

**Conclusion:**

These two case reports and the literature review emphasize the pathophysiological mechanisms leading to potentially devastating complications. In order to reduce the risk of vision loss secondary to cosmetic filler injections, practitioners should possess a thorough knowledge of anatomy and preventive strategies.

**Supplementary Information:**

The online version contains supplementary material available at 10.1186/s42466-022-00203-x.

## Introduction

There is an ongoing expansion of the cosmetic armamentarium promising rapid and simple facial rejuvenation with long-lasting results. A growing number of cosmetic procedures are performed by non-medical practitioners, suggesting risk-freeness. In addition, easy access to the procedures can outweigh judgement of the level of qualification necessary to conduct these interventions safely. Risks might be greater than anticipated by the persons undergoing such procedures [1]. Informed consent to all risks of cosmetic filler injections and potential sequelae, including ocular or neurological complications, should be mandatory [2].

A broad spectrum of complications, ranging from mild or transient adverse events to permanent vision loss, is associated with these procedures, as illustrated by two case reports of complications following facial hyaluronic acid filler injections.

## Case 1

A 43-year-old woman was admitted to hospital with monocular vision loss of the left eye in close temporal relation to a hyaluronic acid (HA) filler injection into the glabellar area. Immediately after receiving the injection, the patient perceived an abrupt fogging to complete blackness of the left eye as well as increased pressure and pain. The non-medical practitioner initiated an ocular massage and waited for the symptoms to resolve. However, her symptoms did not improve, and the patient presented to the emergency room one hour later accordingly. The ophthalmological examination revealed a left relative afferent pupillary defect (RAPD). On funduscopic examination, the optic disc showed sharp margins, thinned arteries and retinal whitening. Initially, no conjunctival injections or vitreous opacities were identified. An ophthalmic artery occlusion (OAO) was diagnosed upon identification of a “cherry-red spot”. The patient was referred to our neurology department to evaluate further treatment options. On initial examination 3.5 h after the onset of symptoms, the patient presented with monocular vision loss of the left eye, ptosis, ophthalmoplegia, periocular pain and nausea, cutaneous changes of the glabella region and forehead and sensory impairment in the left maxillary branch dermatome (V2). Peripheral reflexes and the remainder of the neurological and physical examinations were normal. She was devoid of a past medical history and was not taking any medications. She complained that the injecting non-medical practitioner (in German: “Heilpraktiker”; non-medical practitioners offer a broad range of complementary and alternative methods in Germany) [3, 4] failed to outline the risks of the procedure when obtaining informed consent.

### Cerebral imaging

**Fig. 1:**
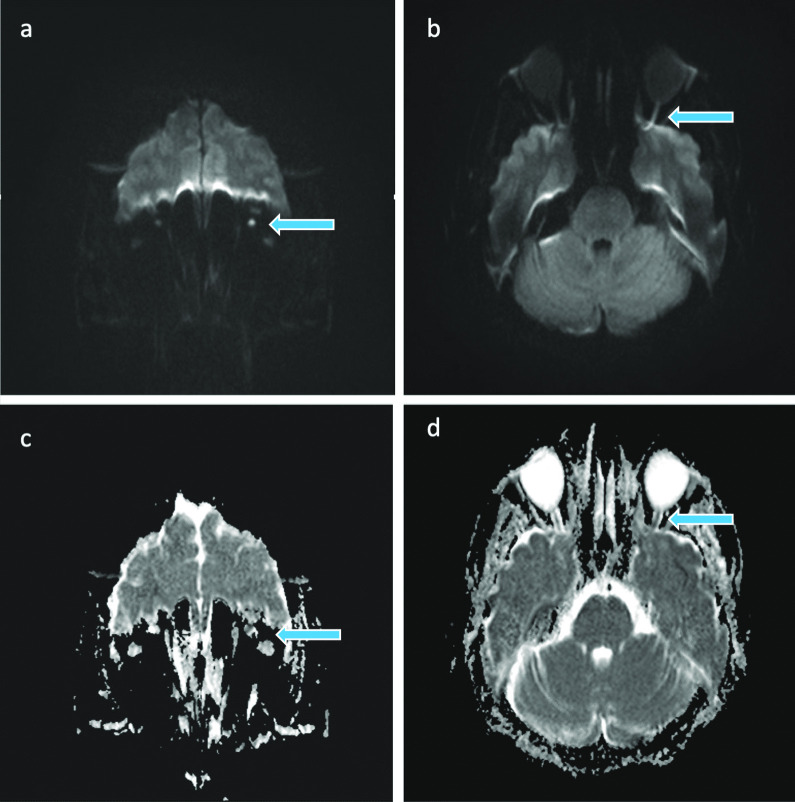
Initial MRI of the brain. Coronal (**a**) and axial (**b**) diffusion weighted imaging (DWI) b = 1000 images and corresponding apparent diffusion coefficient (ADC) maps (**c, d**) depicting high b = 1000 DWI signal and low ADC in the left optic nerve of our patient at presentation

**Fig. 2:**
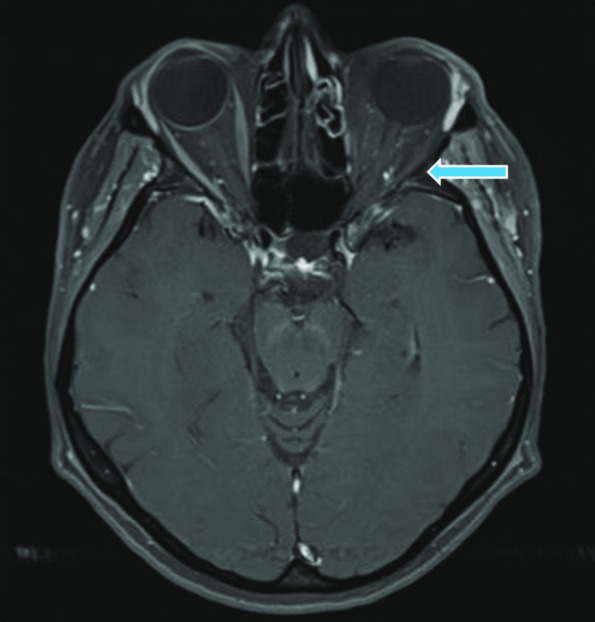
Post-contrast, axial T1-weighted fat-saturated image at presentation. The orbital structures, including the ocular bulb and the rectus muscles on the left side, show no contrast enhancement indicating an ischemic process

The initiated brain MRI revealed an ischemic optic neuropathy on the left side without evidence of a thrombosis in the cavernous sinus (Figs. 1, 2). There was no evidence of further brain infarction on imaging.

### Emergency management of visual loss secondary to cosmetic filler injection

Due to a lack of available evidence-based treatments, two supportive therapies consisting of either off-label use of intravenous thrombolysis with recombinant tissue plasminogen activator (rtPA) or the administration of aspirin, heparin, and systemic steroids were discussed in detail with the patient. The risk–benefit ratio of administering systemic thrombolysis was outlined, including the risk of symptomatic haemorrhage at the impending skin necrosis sites culminating in facial scar formation. An alternative and more conservative treatment option was offered, including aspirin, steroids, and heparin combined with local treatment, as this had been shown to result in a slight improvement in some cases [5]. The patient consented to the latter treatment and, thus, received aspirin (100 mg, once per day, orally), steroids (methylprednisolone 100 mg, once per day, intravenously), heparin (tinzaparin sodium injection 4,500 IU, once per day, subcutaneously) and hyaluronidase injections into the skin at the initial injection site on the following days.

### Follow-up

**Fig. 3:**
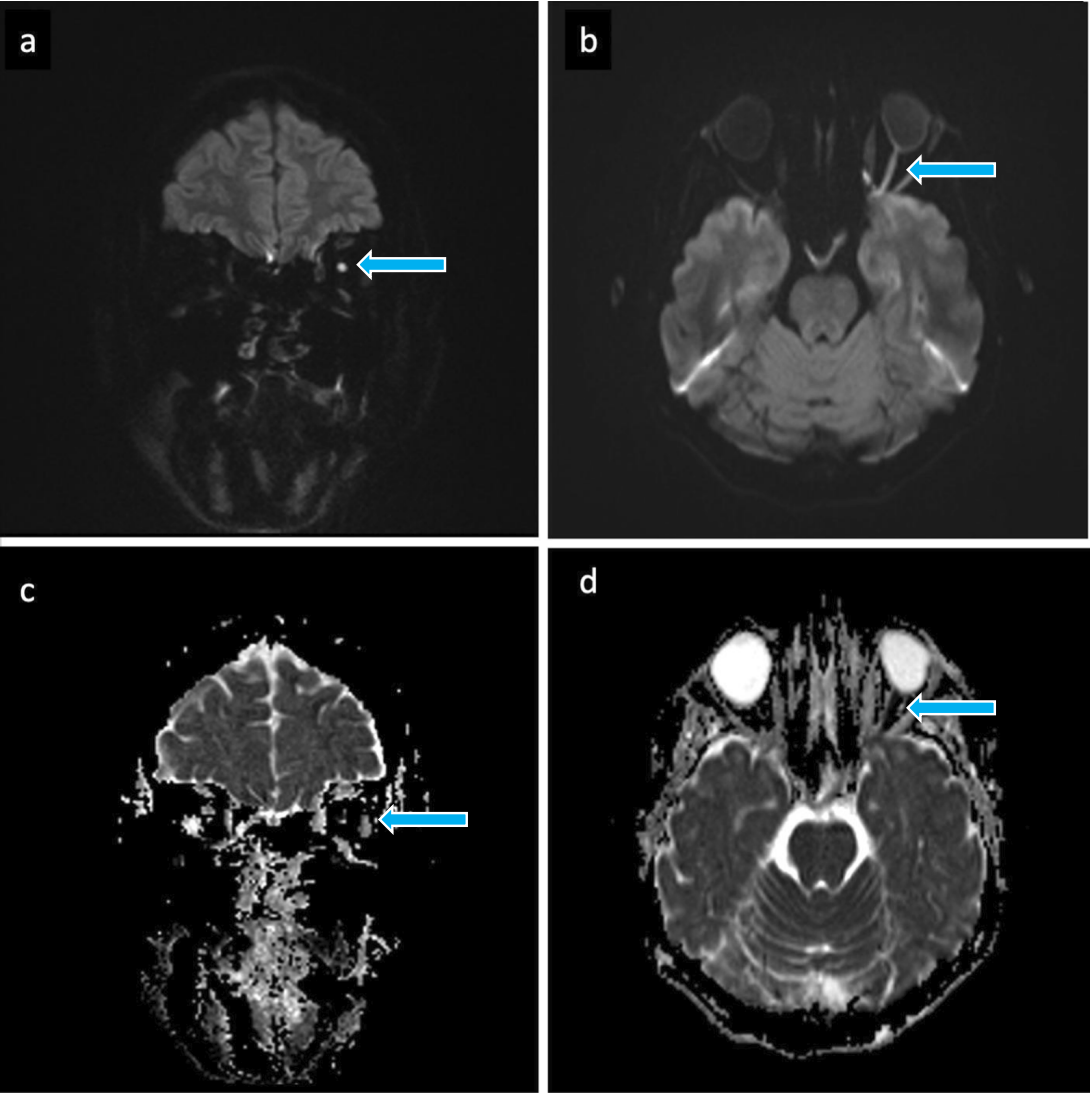
MRI of the brain 1.5 weeks after the HA filler injection. Coronal (**a**) and axial (**b**) diffusion weighted imaging (DWI) b = 1000 images and corresponding apparent diffusion coefficient (ADC) maps (**c**, **d**) highlighting more pronounced signal changes in the left optic nerve

**Fig. 4:**
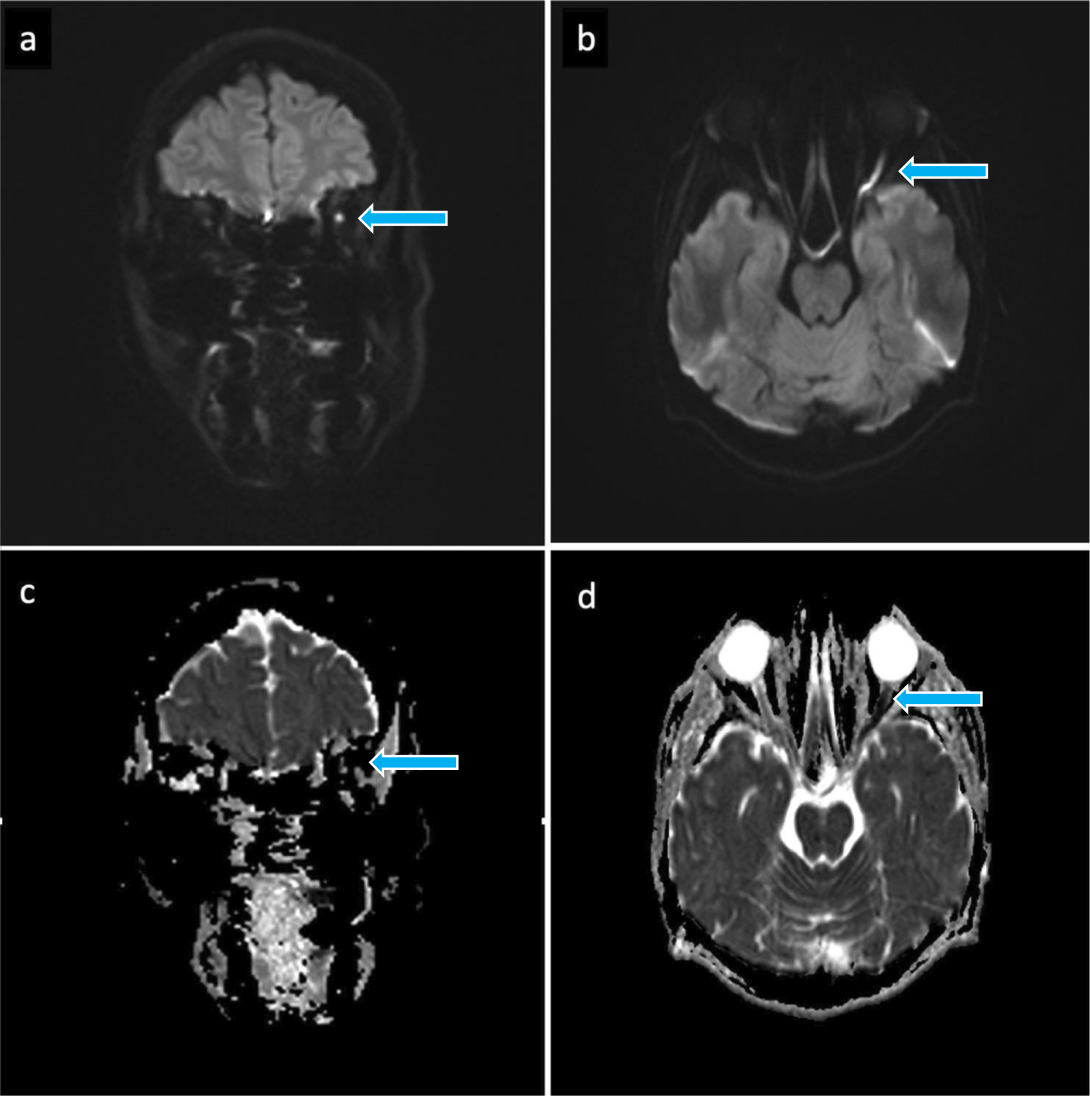
MRI of the brain two months after the HA filler injection. Coronal (**a**) and axial (**b**) diffusion weighted imaging (DWI) b = 1000 images and corresponding apparent diffusion coefficient (ADC) maps (**c, d**) corresponding to stable MRI findings

Follow-up MRIs of the head performed 1.5 weeks and two months after presentation corresponded to stable MRI findings (Figs. 3, 4). There was a significant improvement in ophthalmoplegia; however, disturbances in ocular motility persisted. In addition, the patient developed enophthalmos and had irreversible and complete vision loss in the left eye. The ptosis had resolved.

## Case 2

**Fig. 5:**
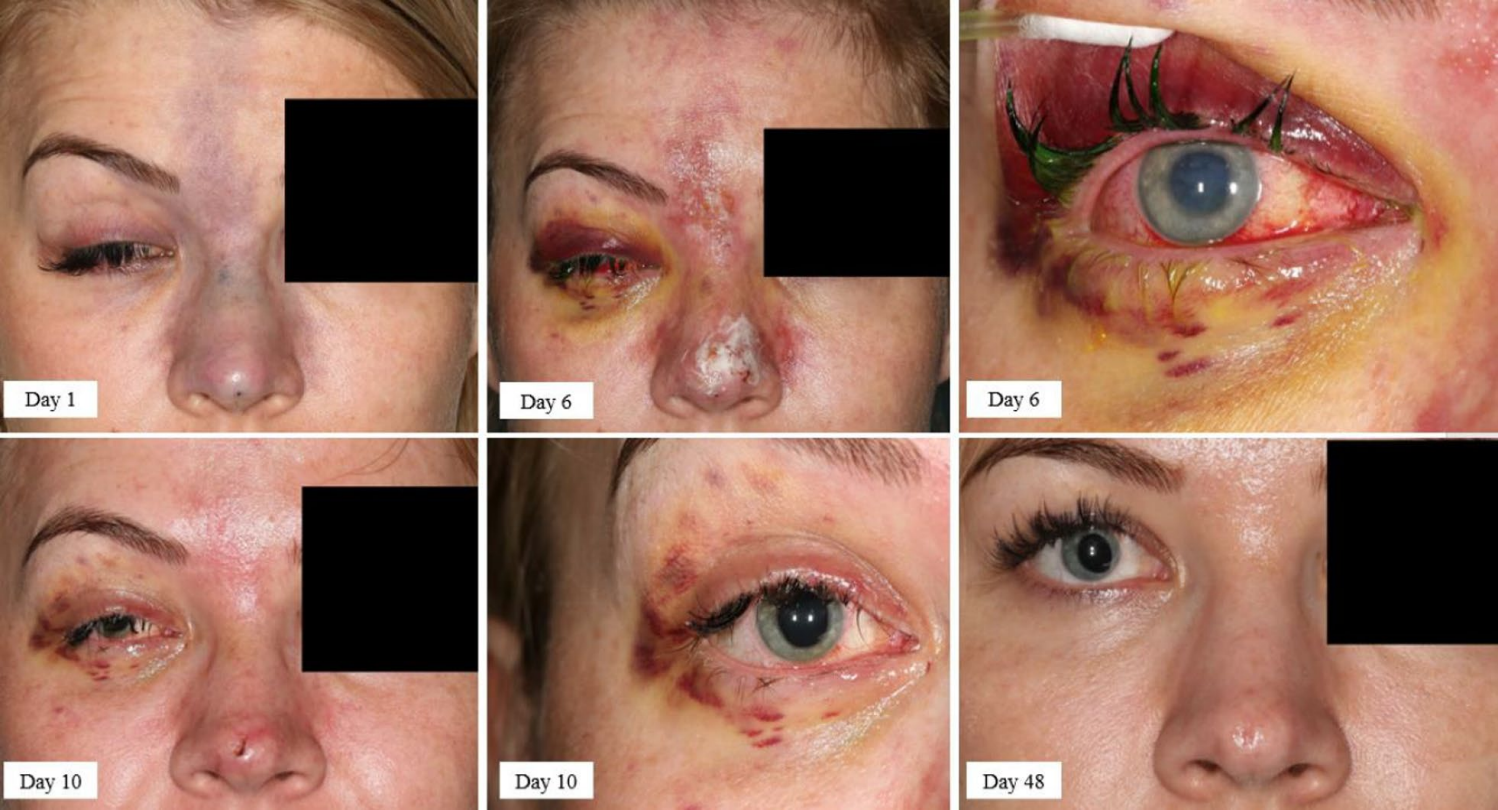
Early and late macroscopic findings following rhinoplasty and cheek augmentation with a hyaluronic acid filler. The patient is shown on post-injection days 1, 6, 10 and 48, demonstrating the oculomotor nerve palsy on the right side and skin necrosis beginning. A small scar at the tip of the nose and an irregular pupillary margin on the right side persisted (day 48)

A 29-year-old woman was admitted to hospital several hours after a rhinoplasty and cheek augmentation with HA. Immediately after receiving the injection, the patient experienced acute monocular vision loss in the right eye, retrobulbar pain, fatigue, and vomiting. Initial examination of the patient revealed a harbinger of impending skin necrosis, as shown in Fig. 5. Additionally, the patient presented with a complete oculomotor nerve palsy on the right side, choroidal ischemia and vision impairment (0.3 cum correctione sua (ccs)).

A brain MRI, fluorescein angiography and further interdisciplinary consultations including neurology, ophthalmology, and dermatology were performed.

The patient was treated with systemic antibiotic therapy with ampicillin/sulbactam, high-dose methylprednisolone and low molecular weight heparin. In addition, several hyaluronidase injections into the ischemic tissue, including the nose and forehead, and sub-conjunctival dexamethasone injections were administered over the following days.

### Follow-up

The long-term follow-up revealed that the skin changes and eye perfusion abnormalities were only partially resolved. A small scar at the tip of the nose, vision impairment (0,8 ccs) and an irregular pupillary margin on the right side persisted.

## Pathophysiological mechanisms

The high molecular weight polysaccharide HA was first described in 1934 by Karl Meyer and John Palmer in bovine vitreous humour [6]. Since then, HA has been discovered in various tissues in humans and vertebrates. Various chemical formulations and application areas have been identified.

Injectable dermal fillers for cosmetic indications, i.e. noninvasive facial rejuvenation, are very popular and commonly considered safe. Their availability and composition may explain their popularity. However, potential devastating complications and long-term sequelae may be underestimated.

Von Bahr published a case report on multiple embolisms in the fundus of the eye after injection into the scalp with hydrocortisone in 1963, stating that “retinal ischemia secondary to embolism is so rare that the risk is not generally realized by doctors” [7]. Meanwhile, studies have identified a rising number of cases and have reflected on best management practices to prevent irreversible deficits. Globally, at least 146 cases of vision impairment secondary to filler treatments have been reported prior to September 2018 [5]. Due to its complex anastomotic network, all face regions are at risk of this complication [8–12]. Kapoor et al. presented the anatomical area distribution in post-HA filler vision loss cases, with the glabella represented in the top three danger zones of sole injection sites next to the nose and forehead [13].

On behalf of a national survey by the Korean Retina Society on iatrogenic vascular occlusion due to cosmetic filler injections, and based on fundus photographs and angiographic findings, ophthalmic artery occlusion was classified into six types: (1) ophthalmic artery occlusion (OAO), as diagnosed in Case 1, (2) generalized posterior ciliary artery occlusion (PCAO) with relative central retinal artery sparing, (3) central retinal artery occlusion (CRAO), (4) localized PCAO, (5) branch retinal artery occlusion, and (6) posterior ischemic optic neuropathy (PION) [14].

The previously healthy 43-year-old woman in Case 1 reported a sudden and permanent vision loss immediately after HA injection, presuming a purely mechanical vascular obstruction. While the diagnostic path from a neurological point of view is normally based on detecting large artery atherosclerosis, embolism (from the heart, aorta or great vessels) or inflammatory vascular disease leading to interrupted blood flow to the retina, the exact underlying pathophysiology of HA complications often remains a conundrum.

The predominant hypothesis is based on a retrograde flow of HA filler supported by accidental cannulation of the supratrochlear artery (intra-arterial injection) with an injection pressure exceeding systolic pressure, followed by a subsequent anterograde flow of HA filler leading to vascular obstruction [15–17]. The proposed mechanism was investigated by Cho et al. when implementing a new cadaver perfusion technique [18] and confirming the pathophysiology of filler-induced blindness.

Beleznay et al. proposed that the associated ptosis results from reduced blood supply to the levator palpebrae superior muscle or its innervating nerves, while ophthalmoplegia could also be caused by the mere obstruction of the blood supply to the extraocular muscles [5]. In addition, nausea and vomiting could occur secondary to increased intraocular pressure [5]. In most reported cases, ophthalmoplegia and lid ptosis commonly recovered, while the retinal damage and subsequent vision impairment were preserved, indicating the possibility of neuromuscular regeneration occurring after vascular compromise [5].

In our case, the patient presented with additional sensory impairment in the left maxillary branch dermatome (V2). From the neurologist’s perspective, the first differential diagnosis included a potential cavernous-sinus involvement, explaining the affected constellation of the vasa nervosa of the oculomotor nerve, the trochlear nerve, the abducens nerve, and the maxillary nerve. However, this would not explain the absence of involvement of the ophthalmic branch (V1), which also traverses through the lateral wall of the cavernous sinus.

An alternative explanation for the involvement of the left V2 dermatome would be a retrograde embolism, distributed from the injection site at the glabella into the infraorbital artery located in anatomical proximity to the V2 branch of the infraorbital foramen.

Additional studies have shown that the vessel volume of the supratrochlear artery from the glabella to the orbital apex is 85 µL, and one should not inject more than this critical volume as a bolus [19]. Non-invasive mapping of facial vascularization revealed high peak systolic velocities re-enforcing the embolic nature of this mechanism and leading to the immediate onset of symptoms [20]. Furthermore, it is important to be cautious with regard to injection pressure [21]. As soon as the injection pressure exceeds systolic pressure, the risk of retrograde embolism and ocular embolization is increased [9, 22] (Additional file 1: Table 1).

## Discussion of management

Transient experimental occlusion of the central artery of the retina in rhesus monkeys lasting up to 98 min did not produce significant permanent neural damage. However, occlusion for 105 min or longer resulted in irreversible damage underlining the urgency of initiating swift treatment [23]. Kapoor et al. reviewed 26 articles, including details of 44 cases. Six of these cases received intra-arterial hyaluronidase two to ten hours after HA filler-induced vision loss and showed no improvement [13]. Wang et al. retrospectively reviewed 30 patients with HA filler-induced monocular blindness treated with intra-arterial injections of hyaluronidase and mechanical recanalization. Among these patients, 12 were treated within 48 h, showing visual improvement in four cases, while only one patient with increased visual acuity was observed among those treated at later time points [24]. Nguyen et al. combined intra-arterial hyaluronidase administration with additional thrombolytic agents (alteplase) in one case, achieving significant vision recovery [25].

Intra-arterial obstruction is regarded as the main reason for skin necrosis [26–36], meaning that the application of hyaluronidase may improve impending necrosis when applied early. It is appropriate to involve ophthalmologists, dermatologists and plastic surgeons in this process. Local allergic reactions represent potential side effects of hyaluronidase.

Even though the efficacy and safety of aspirin and heparin have not been proven in hyaluronic acid related occlusions, studies have highlighted their potential of preventing thrombotic spreading [17, 30, 37–42]. The rationale behind the application of aspirin is based on extrapolation from other clinical settings, i.e. evidence from acute coronary syndrome and ischemic stroke [43–45].

While Murray et al. urge clinicians to consider potential risks of instituting steroid therapy, i.e. impaired wound healing and increased risk of infection [43], most studies acknowledge the potential of decreasing the inflammatory component of the injury [5, 37, 40, 42, 46].

## Conclusions

The Aesthetic Complications Expert (ACE) Group identified several key preventive strategies aimed at minimizing the risk of vision loss secondary to cosmetic filler injections, including a careful low injection pressure, injections of small increments and aspiration [8]. Non-medical practitioners should possess a thorough knowledge of anatomy and preventive strategies. In case of adverse events after injection, non-medical practitioners should be aware of urgent and interdisciplinary management strategies and refer their patients swiftly for appropriate medical treatment.

## Supplementary Information

**Additional file 1:** Suggestions for potential medical standard operating procedures.

## Data Availability

Data transparency.

## Abbreviations

ACE: Aesthetic complications expert
ADC: Apparent diffusion coefficient
ccs: Cum correctione sua
CRAO: Central retinal artery occlusion
DWI: Diffusion weighted imaging
HA: Hyaluronic acid
MRI: Magnetic resonance imaging
OAO: Ophthalmic artery occlusion
PCAO: Posterior ciliary artery occlusion
PION: Posterior ischemic optic neuropathy
rtPA: Recombinant tissue plasminogen activator
