# Supplementary material for: Sudden vision loss and neurological deficits after facial hyaluronic acid filler injection
Source: Neurol Res Pract. 2022 Jul 18;4:40. doi: 10.1186/s42466-022-00203-x (PMC9290300; doi:10.1186/s42466-022-00203-x)
Supplement: Supplementary file 1 — Additional file 1: Suggestions for potential medical standard operating procedures. [file 42466_2022_203_MOESM1_ESM.docx]

Clinical Pathway: **Time is vision**!

| Immediate transfer of symptomatic patient to hospital setting, i.e. interdisciplinary emergency department, including a specialist eye unit. |
| --- |
| Check vital signs. Continuous monitoring is recommended. |
| Management of ocular pain or headache as well as nausea and vomiting, as these are common symptoms. |
| Immediate evaluation by an ophthalmologist. Document vision changes and further pathologies. Confirm diagnosis. |
| Consider injecting hyaluronidase via a retrobulbar or peribulbar application by practitioners competent in this procedure. (PMID: 28333326) |
| Assessment by a neurologist. Perform a magnetic resonance imaging (MRI) scan of the brain to rule out infarction. |
| Consider thrombolytic agents (alteplase)/intra-arterial hyaluronidase/mechanical recanalization. (PMID: 28333326; PMID: 34767060) Alternatively, start treatment with aspirin, steroids and heparin. |
| Evaluation by a dermatologist/plastic surgeon and repeated administration of hyaluronidase into the ischemic tissue. |

**Supplementary Table 1**. Suggestions for potential medical standard operating procedures.
